# Thyroid Screening Techniques via Bioelectromagnetic Sensing: Imaging Models and Analytical and Computational Methods

**DOI:** 10.3390/s24186104

**Published:** 2024-09-21

**Authors:** Anna A. Varvari, Alexandros Pitilakis, Dimitrios I. Karatzidis, Nikolaos V. Kantartzis

**Affiliations:** School of Electrical and Computer Engineering, Aristotle University of Thessaloniki, 54124 Thessaloniki, Greece; avarvari@ece.auth.gr (A.A.V.); karatzidis@ece.auth.gr (D.I.K.); kant@auth.gr (N.V.K.)

**Keywords:** bioelectromagnetic sensing, computational electromagnetics, electromagnetics-based techniques for disease localization and classification, imaging models, thyroid screening methods

## Abstract

The thyroid gland, which is sensitive to electromagnetic radiation, plays a crucial role in the regulation of the hormonal levels of the human body. Biosensors, on the other hand, are essential to access information and derive metrics about the condition of the thyroid by means of of non-invasive techniques. This paper provides a systematic overview of the recent literature on bioelectromagnetic models and methods designed specifically for the study of the thyroid. The survey, which was conducted within the scope of the radiation transmitter–thyroid model–sensor system, is centered around the following three primary axes: the bands of the frequency spectrum taken into account, the design of the model, and the methodology and/or algorithm. Our review highlights the areas of specialization and underscores the limitations of each model, including its time, memory, and resource requirements, as well as its performance. In this manner, this specific work may offer guidance throughout the selection process of a bioelectromagnetic model of the thyroid, as well as a technique for its analysis based on the available resources and the specific parameters of the electromagnetic problem under consideration.

## 1. Introduction

The thyroid is decisive for the regulation of hormonal levels and drastically impacts the function of other organs. As our exposure to electromagnetic radiation increases daily, driven by the rapid development of wireless technologies, it is evident that scientific research on the possible side effects on the thyroid gland is also constantly expanding [1,2,3,4,5,6,7,8,9,10,11,12]. Furthermore, a variety of thyroid models has been developed in recent years to support attempts at representing the interaction of the thyroid with devices, improving screening techniques, and detecting diseases such as cancer [13,14,15,16,17]. Within this framework, we introduce the thyroid sensor system and its variations. We then discuss the diverse models of the thyroid, categorized according to their characteristics, and provide a summary of the methods used for the analysis of these models.

**Figure 1:**
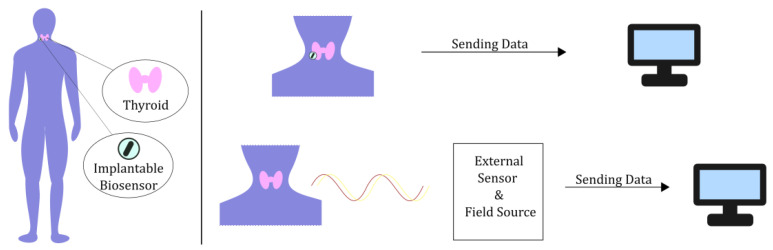
General representation of the thyroid sensor system.

**Figure 2:**
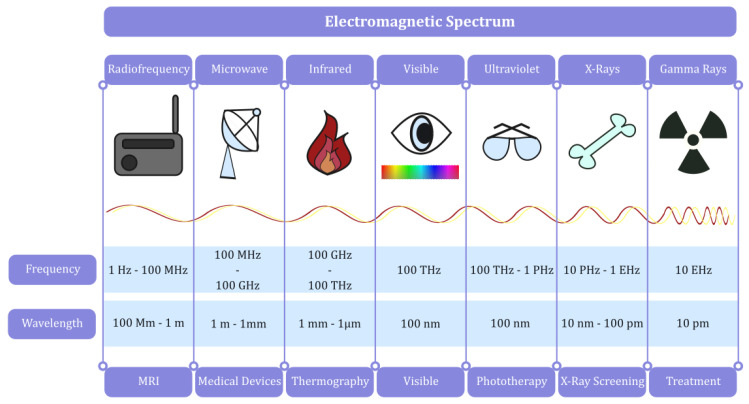
The electromagnetic spectrum (frequency and wavelength expressed in orders of magnitude).

Whether it is for diagnostic, therapeutic, or even monitoring purposes [18,19,20,21,22], the system under study always takes the same form, as depicted in Figure 1. A sensor that detects electromagnetic radiation is placed either on/in the thyroid or a relatively short distance from it. In some cases, such as in magnetic resonance imaging (MRI), a source of radiation is also included so that the sensor can measure the scattered radiation. It should be noted that when the nature of the data is such that no conclusion with respect to the measurement can be derived using just the computational power of the system (e.g., a plain thermal camera), then an external processor is needed, like a computer device, etc. In this context, the aforementioned system may be used in screening and digital imaging (e.g., thermography or volumetry) and in radiation detection/ablation for both diagnostic and therapeutic purposes. Actually, the applications may vary, depending on the implementation method and the band of the frequency spectrum used, as described in Figure 2 and Table 1.

More elaborately, we focus particular attention on the lower half of the frequency spectrum, focusing on the radio frequency (RF) and infrared spectrum. Most wireless technologies used in everyday life operate in a specific spectrum, raising concerns about potential hazards and effects on the thyroid. Moreover, microwaves may be needed for several medical devices (e.g., parts of a linear accelerator or diagnostic radar), while the infrared region is used in thermography. Finally, the upper half of the frequency spectrum is mainly used for therapeutic and diagnostic purposes. The various (electromagnetic) screening tools and techniques for the thyroid, along with their principal characteristics and most popular applications, are listed in Table 1.

It is worth mentioning the specific case of biosensors in the form of antennas. Their distinguished feature is their adaptability to various scenarios, as they can be implanted and inserted in the thyroid but also be used as wearables or simply placed on the surface of the neck [23,24,25,26,27,28,29,30,31]. Actually, the majority of antennas that must be positioned close to the thyroid or on the surface of the neck [24,25], as well as of wearable antennas [26,27,28,29], operate in the radio frequency (RF) spectrum. There are, however, antennas in the form of probes that must be set on or inserted in the thyroid [30,31], which are mainly used for temperature monitoring purposes during thyroid ablation.

In this survey, we provide a comprehensive review of the bibliography, which focuses on models and methods developed for the representation of the human thyroid. Particular attention is paid to thermographic, 3D volumetric, and radiation models, which simulate the response of thyroid tissues to exposure to electromagnetic radiation or heat, as well as the most significant analytical, computational, artificial intelligence, and machine learning techniques for the rigorous solution of the corresponding electromagnetic problem.

## 2. Models and Techniques

### 2.1. Bioelectromagnetic Models for Medical Imaging Applied to the Thyroid

The vast majority of thyroid models are either based on the collection of data with screening techniques, followed by statistical analysis, or derived as voxel data from a single screening. Evidently, these models can, also be abstract representations of the thyroid, which act as faster methods for deriving an approximation of the object under research (e.g., the intensity of the electromagnetic field in the tissue). In all cases, whether realistic or approximate, the aforementioned bioelectromagnetic models are deemed powerful tools in the study of the thyroid and are required for simulations of radiation–thyroid interactions.

Furthermore, when studying thyroid status, the following three key factors must be taken into consideration: the shape, the temperature, and the tissue condition. The shape and temperature of the thyroid may provide adequate information concerning the condition of its tissues, although without yielding the complete image and being able to determine the radiation absorption in each case. Therefore, we divide bioelectromagnetic models into the following three main categories: volumetric models, thermographic models, and models concentrating on the measurement of the radiation or absorption/ablation levels. These models are more thoroughly presented below.

#### 2.1.1. Volumetric Models

The accurate estimation of the thyroid’s volume is critical for therapeutic invasive (e.g., surgery) and non-invasive (e.g., radiation ablation) procedures, as well s for diagnostic purposes [32]. Additionally, there is a significant correlation between the thyroid volume and anthropometric indices in general [33]. Therefore, volumetric data are of great importance and may prove valuable for the treatment of a patient.

On the surface level, the fast approximation of the thyroid size provided by ultrasound may be very useful; however, it lacks the necessary resolution. Hence, even though ultrasound is the main technique used in the field of volumetry, volumetric data may be obtained through other screening techniques, such as MRI or CT, or even via thermal images in the infrared spectrum [32,34,35,36] following the construction of a 3D reference model. In fact, the selection of a method for the construction of a thyroid model depends on the size/magnitude and quality of the obtained data, as well as the purpose of the model (i.e., patient-specific reference model [37] or a generic model). Furthermore, the fusion of several images is possible [38] in cases in which higher accuracy or extra information is required [38].

The design method explored in this section is the Structure from Motion (SfM) [34] model, in which multiple 2D CT images refer to different viewpoints of the thyroid, allowing for the extraction of thyroid measurements in 3D. Similarly, multiple 2D scintigraphy images may lead to the same result, taking into consideration the anatomical prior in order to compensate for the lack of knowledge regarding the third dimension [35]. Moreover, in most cases, a statistical analysis must follow to create a more precise 3D representation of the human thyroid.

**Figure 3:**
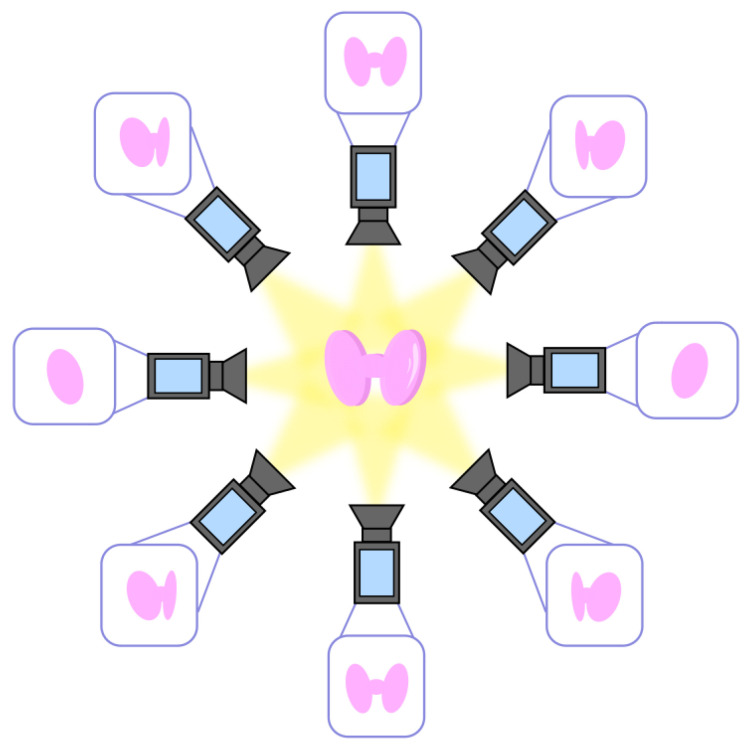
Multiple 2D images from different angles.

Specifically, the SfM method can be described by the following steps:

- *Step 1: Multiple 2D images*. Extraction of 2D images of the thyroid from multiple angles, as qualitatively depicted in Figure 3;
- *Step 2: Point correspondences*. The point correspondences from multiple images are called tracks and are associated with 3D points of the thyroid. The computation of the tracks can be performed pairwise for two consecutive camera angles until all are fully described;
- *Step 3: Construction of the 3D model*. The data are combined to create a 3D coordinate system, which leads to the complete 3D model of the thyroid.

#### 2.1.2. Thermographic Models

Similar to volumetric data, the thyroid’s temperature map can provide useful information for the diagnosis of disease [39,40,41]. Even though thermal images can be obtained using MRI or CT scans, another popular way to obtain thermographic data is via thermal cameras and sensors [42]. In this context, the selection of the appropriate solid-state sensor depends on a variety of parameters, as summarized in Table 2. In particular, thermocouples are used for wide temperature ranges, yet they have been proven to be the least stable. On the other hand, resistance temperature detectors (RTDs) are sufficiently more linear and stable, but their usage, such as in highly sensitive thermistors, can lead to self-heating. Finally, liquid crystal sensors are preferred for their simplicity and durability, although their temperature range is rather limited [42,43].

Using the abovementioned screening techniques and sensors, it is possible to reconstruct a temperature map of the thyroid [34,37,38,44,45]. As in volumetry, the reconstruction of the model may involve statistical analysis or even the use of artificial neural networks [32,40] when it comes to generic cases. Still, for patient-specific thermal mapping, no additional steps are required [37]. In some cases, however, the fusion of several images obtained by means of various screening techniques may be instructive for the combination of volumetry with thermographic data, increasing the overall accuracy [38]. Additionally, thermographic models of the thyroid can be designed to be realistic or simple representations of basic shapes, such as a half disk for the neck with internal lobes, where the thermal characteristics of the tissues are the same as those of the corresponding shapes [40,42,43,45,46,47]. Note that since the majority of these models are purely computational, they can be effectively utilized in the determination of electromagnetic radiation levels.

#### 2.1.3. Radiation Detection/Ablation Models

The study of radiation distribution and absorption by the thyroid tissues and their dielectric properties bares great significance, as the thyroid appears to be highly sensitive concerning exposure to radiation [11,48,49]. Furthermore, apart from scanning and diagnosis, radiation detection is undoubtedly a necessary step when it comes to therapeutic procedures that deploy radiation ablation. Even though radiation models can be constructed similarly and are intertwined with those intended for volumetry and thermography (therefore, also providing the basis for volumetric and thermal analysis), they may serve as valuable tools for the measurement of radiation levels in tissues.

Analogously to volumetry and thermography, thyroid models for radiation detection are (semi-)realistic, acquired by screening techniques and recombination of data or they simplified via the use of basic shapes (like a cylinder or sphere for the neck and ellipsoids for the lobes) [28,46,50,51]. The latter can also be employed for the evaluation of the temperature distribution, typically through the FEM [46,51,52,53].

When it comes to the RF spectrum, the most important metric concerning radiation absorption is the specific absorption rate (SAR), which is the rate at which energy is absorbed per unit mass and may prove instructive in diagnostics [25,26,27,46,50,51,54,55]. Generally, for a sample of tissue, the SAR is defined as

(1) $$\mathrm{SAR}=\frac{1}{V}\int_{V}\frac{\sigma \left(r\right)E^{2}\left(r\right)}{\rho (r)}\;dV\;\left(W/\mathrm{kg}\right),$$

where σ is the electric conductivity (S/m), E is the electric-field intensity vector (V/m), ρ is the density (kg/m^3^), and *V* is the volume of the sample (m^3^).

#### 2.1.4. Model Attributes and Application Scope

Each one of the three model categories (volumetric, thermographic, and radiation) has its own characteristics and application scope.

In more detail, in the majority of cases, volumetric models represent the thyroid by means of a mesh. The shape, the volume, and the particular attributes of the thyroid surface are considered accordingly. It should be stressed that volumetry is proven to be very helpful for reference models, especially in patient-specific scenarios, when an invasive operation is planned or when a more sound comprehension of the size and the location of the thyroid is required. They can also constitute an instructive and trustworthy diagnostic tool for a variety of thyroid deceases, since, in some applications, the shape or the size of the thyroid nodules might be affected by them [32,34,35,36].

Apart from volumetric models, thermographic models can serve as temperature maps of the thyroid with increased levels of reliability. In fact, these models have been found to be extremely helpful in assessing the response and sensitivity of the thyroid to electromagnetic fields; hence, they have monitoring purposes in procedures such as radiation ablation therapy. Note that the temperature of the thyroid is also affected by deceases; therefore, these models may be employed for diagnostic objectives [37,43,46].

Finally, radiation detection/ablation models are maps of the dielectric properties of the thyroid and can be deemed volumetric and thermographic models as well, with the right adaptations. However, their key goal is the accurate measurement of radiation levels in the thyroid, which renders them ideal for monitoring purposes in radiation therapy. The detection of radiation, together with its potential origins, can, furthermore, assist in the assessment of the impact of radiation on the thyroid, since the majority of the models can be used as a reference for an assortment of radiation types [27,50,51,52].

#### 2.1.5. Statistical Classification of the Related Bibliography

**Figure 4:**
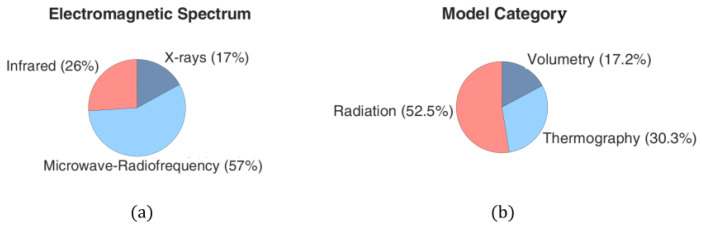
Publication percentages based on (**a**) the frequency spectrum considered for thyroid screening and (**b**) the method of analysis.

**Figure 5:**
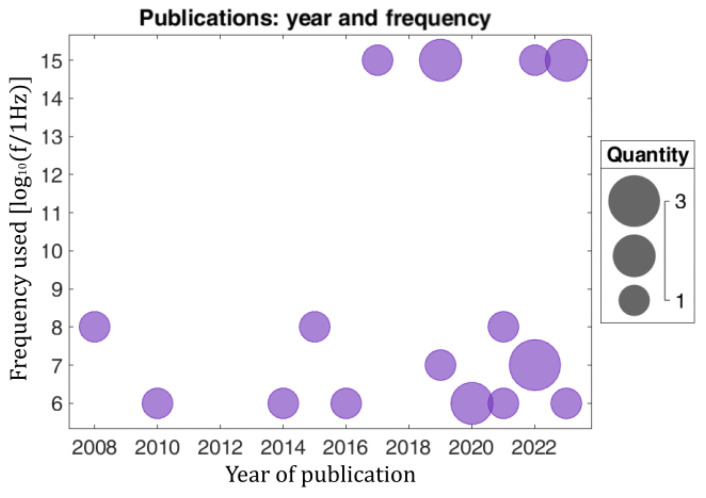
Number of relevant publications for every year versus the employed frequency band in MHz on the X-ray spectrum.

Having described the most important methodologies, in this subsection, we provide some general and interesting statistical results categorized according to the characteristics of the computational models that were analyzed in twenty highly relevant publications published during the temporal interval of 2008–2023. For the sake of convenience, these data are summarized in Table 3, Figure 4 and Figure 5.

### 2.2. Models for the Analysis of Bioelectromagnetic Data

#### 2.2.1. Analytical Methods

**Figure 6:**
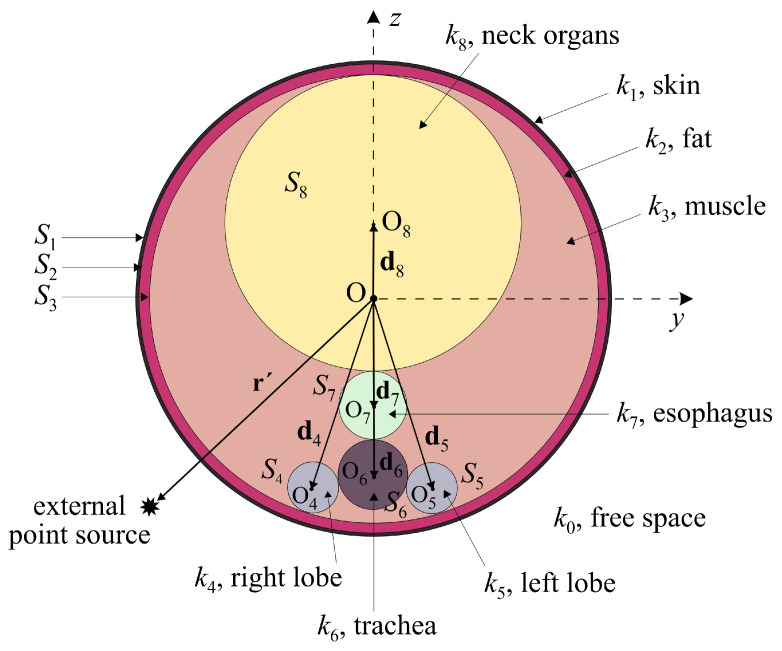
Cross-section of the non-spherical thyroid model.

An intuitive and versatile tool that is significantly economical in terms of computational (CPU time and memory) resources can be pursued in the development of appropriate analytical models of the human thyroid. These quantitative formulations are abstract and simple enough to be consistently defined and rapidly solved [56,57]. To this end, let us consider the non-spherical model presented in Figure 6, comprising a main sphere with two spherical layers representing skin ($S_{1}$) and fat ($S_{2}$), while a third cocentricstructure describes muscle tissue ($S_{3}$). Moreover, the model involves non-overlapping spheres representing the right thyroid lobe ($S_{4}$), the left thyroid lobe ($S_{5}$), the trachea ($S_{6}$), the esophagus ($S_{7}$), and the other neck tissues ($S_{8}$). Probing further, every distinct sphere has a radius of $a_{i}$ for i=1,2,…,8, with the cocentricones centered at the origin (O) and the eccentric ones at $O_{j}$, denoted by vectors ($d_{j}$) for j=4,5,…,8. It is stressed that all parts contain a lossy dielectric material with a complex relative permittivity and a wave number of $k_{i}$ for i=1,2,…,8.

Assuming a harmonic time dependence, the specific analytical method uses the dyadic Green function (dGf) for the solution of the resulting system of equations. Explicitly, the source is considered an ideal Hertz dipole (point source) so that the free-space values of the electric field can be obtained through the dyadic Helmholtz equation as follows:

(2) $$\nabla \times \nabla \times {\bar{\bar{G}}}_{e,s}^{\left(0\right)}-k_{0}^{2}=\bar{\bar{I}}\delta \left(r-r^{\prime }\right),$$

where ${\bar{\bar{G}}}_{e,s}^{\left(0\right)}$ ia the free-space Green dyad, $k_{0}$ is the free-space wave number, and $\bar{\bar{I}}$ is the unit dyad.

Subsequently, by using the Ohm–Rayleigh method [58], the dGf can be expressed in terms of vector spherical harmonics as

(3) $${\bar{\bar{G}}}_{e,s}^{\left(0\right)}=-\frac{1}{k_{0}^{2}}\hat{r}{\hat{r}}^{\prime }\delta \left(r-r^{\prime }\right)+j\frac{k_{0}}{4\pi }\sum_{nm,a}c_{mn}F_{a,mn}^{\left(1\right)}\left(k_{0}r\right)F_{a,-mn}^{\left(3\right)}\left(k_{0}r^{\prime }\right),$$

with $\hat{r}$ and ${\hat{r}}^{\prime }$ representing the unit vectors of r and $r^{\prime }$, m=−n,−n+1,…,n−1,n, for n=1,2,…, and $c_{mn}={\left(-1\right)}^{m}\left(2n+1\right)/\left[n\left(n+1\right)\right]=c_{-mn}$. Furthermore, if, in (3), we set a=M,N so that $F_{M,mn}^{\left(\iota \right)}=M_{mn}^{\left(\iota \right)}$ and $F_{N,mn}^{\left(\iota \right)}=N_{mn}^{\left(\iota \right)}$, the required spherical vector harmonics are given by

(4) $$F_{M,mn}^{\left(\iota \right)}\left(kr\right)=M_{mn}^{\left(\iota \right)}\left(kr\right)=z_{n}^{\left(\iota \right)}\left(kr\right)\left(jm\frac{P_{m}^{n}\left(\cos\theta \right)}{\sin\theta }\hat{\theta }-\frac{dP_{m}^{n}\left(\cos\theta \right)}{d\theta }\hat{\phi }\right)e^{jm\phi },$$

(5) $$F_{N,mn}^{\left(\iota \right)}\left(kr\right)=N_{mn}^{\left(\iota \right)}\left(kr\right)=\frac{1}{k}\nabla \times M_{mn}^{\left(\iota \right)}\left(kr\right),$$

where $z_{n}^{\left(\iota \right)}\left(kr\right)$ is the first kind of spherical Bessel (for ι=1) or Hankel (for ι=3) function, while $P_{m}^{n}\left(\cos\theta \right)$ is a Legendre function of the first kind. Note that (3) is valid only for $r\leq r^{\prime }$; otherwise, the ι=1,3 superscripts at the spherical harmonics (F(.)) must be reversed.

**Figure 7:**
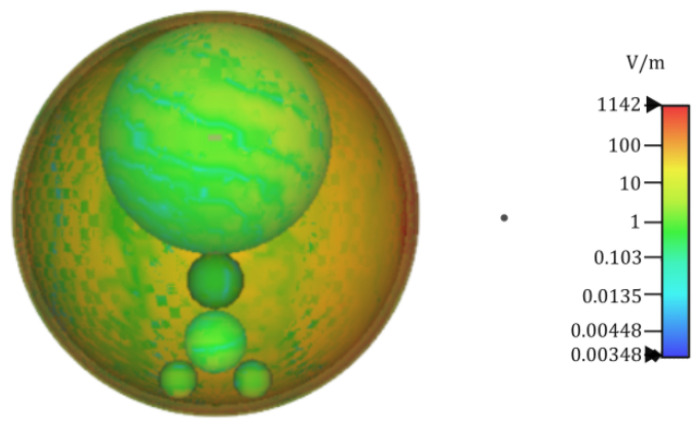
Electric-field intensity map at a cross-section of the thyroid model described in Figure 6 for a point source placed on the right-hand side and marked as a dot. The protective action of the surrounding external skin, fat, and muscle layers against the penetrating electromagnetic waves can be observed.

Based on these aspects, the non-spherical model yields a system of eight equations, where $P_{q}$ (for q=1,2,…,8) is the designated location of the field point of interest in the area confined by two consecutive surfaces. Applying the reciprocity principle and implementing the appropriate boundary conditions, the prior system is solved, and the electric-field intensity at any point ($P_{q}$) is acquired from

(6) $$E^{\left(q\right)}\left(r\right)=j\omega \mu_{0}\underset{V}{\;\int \int \int \;}{{\bar{\bar{G}}}_{e}}^{\left(q\right)}\left(r,r^{\prime }\right)\cdot J^{\left(q\right)}\left(r^{\prime }\right)dV,$$

with *V* representing the limited volume between the two surfaces and $J^{\left(q\right)}\left(r^{\prime }\right)$ representing the electric current density of the external source at $r^{\prime }$. It is interesting to emphasize that such a model can also be analyzed by means of different computational methods for the rigorous derivation of the electric field at every point of the domain [56]. For illustration, Figure 7 presents the distribution of the electric-field intensity in the thyroid model, highlighting the protective role of the external layers (skin, fat, and muscle) against electromagnetic radiation.

#### 2.2.2. Computational Techniques

Apart from analytical schemes, a highly competent and widely acknowledged computational methodology for both the reconstruction and analysis of bioelectromagnetic thyroid models is the finite element method (FEM) [59,60]. In essence, the FEM is a general-purpose numerical technique for the accurate solution of partial differential equations in 2D or 3D spaces. In order to handle a problem, the FEM divides its continuous space into smaller entities (usually triangles or tetrahedrons) called finite elements. This is accomplished in terms of an efficient discretization mechanism realized by the adaptive generation of a lattice, namely the computational domain, which involves a finite amount of properly interconnected nodes. In this way, the FEM formulates every boundary value problem through a system of algebraic equations and approximates all the desired unknown quantities at the nodes of every finite element. Finally, these equations are assembled into a larger system that models the whole problem, whose solution is obtained through the minimization of certain error functions [59]. This can be accomplished either via in-house (developed by the researchers) programming codes or through several highly powerful commercial computational packages, such as COMSOL Multiphysics^®^ simulation software (version 5.4) [61], which can interactively and reliably generate the necessary adaptive thyroid meshes. Hence, regarding, for example, in the analysis of thermographic thyroid data, the FEM provides a temperature map of the object under study, which may refer to a transient state (when heat flux is included) or a static one [42,46,47].

**Figure 8:**
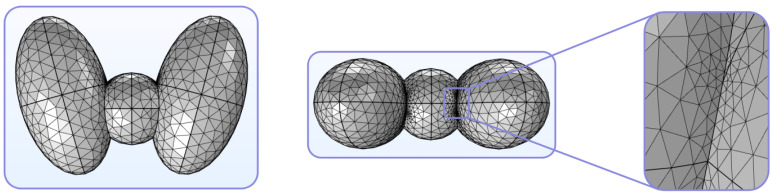
Diverse viewpoints of indicative tetrahedral FEM lattices for an abstract model of the thyroid created using COMSOL Multiphysics^®^ simulation software [61].

In more detail, analysis conducted via the FEM includes the following steps [62]:

- *Step 1: Discretization*. The mesh of the thyroid is constructed, as depicted in Figure 8;
- *Step 2: Selection of shape functions and formulation of the finite element equations*. A pattern is selected for the distribution of the unknown variables, and the suitable governing laws are applied to the general equation of every finite element, i.e.,
  (7) kq=Q,
  where *k* is a square matrix of the characteristics of the homogeneous medium, *q* is a column vector of nodal values (output), and *Q* is a column vector that serves as the excitation (e.g., the field from an illuminating source);
- *Step 3: Assembly of the system of equations*. With the equations of every finite element in the mesh appropriately derived, the global system of equations (modeling the entire problem) is constructed in the general form of
  (8) Kr=R,
  where *K* is the assembly (stiffness) matrix, *r* is the assembly vector of nodal degrees of freedom, and *R* is the assembly vector of nodal forcing parameters;
- *Step 4: Solution of the system*. The linear system of algebraic equations from Step 3 is solved for *r* by means of specific numerical algorithms, such as the incomplete Cholesky conjugate gradient (ICCG) scheme [59,60];
- *Step 5: Interpretation of the results*. The solution of the system leads to an output, which can then be post-processed accordingly in order to calculate several quantities, gauges, or metrics of the model.

In this manner, several challenging thyroid studies can be effectively and meticulously performed without the need for non-physical assumptions or restricting conventions. Essentially, the FEM enables the easier modeling of complex geometrical and irregular thyroid shapes while offering enhanced adaptability to meet certain precision standards and decrease the use of prototype phantom measurements. Lastly, its high levels of accuracy and convergence, combined with several visualization options, provide important means for trustworthy interpretations and consistent deductions from the obtained results.

#### 2.2.3. Artificial Intelligence and Machine Learning

Undoubtedly, the role of artificial intelligence (AI) and machine learning (ML) techniques is anticipated to play a crucial and promising role in the precise analysis and reliable assessment of contemporary bioelectromagnetic problems. Therefore, in this section, we present several emerging ML methods for the manipulation and solution of such problems, which can offer new insights and pave the way in the dynamic processing and fast interpretation of thyroid imaging data. Furthermore, the use of advanced and reconfigurable ML approaches for the systematic modeling of modern biomechanical systems is introduced—a heuristic perspective that could also potentially be employed for bioelectromagnetic models. Essentially, when it comes to the analysis of volumetric [63,64], thermographic [32,40,65], or electromagnetic data [66], several AI algorithms have been, hitherto, presented, with their principal focus being on diagnostics and imaging. Interestingly, these algorithms depend on the datasets obtained from the very same procedures as those utilized for the construction of the thyroid model.

The driving motivation for this conspicuously rapid evolution of AI schemes and ML techniques is the constantly increasing demand for faster and more rigorous results in the field of computational electromagnetics. Furthermore, since the solution of the same problem is typically required, every time an alteration in the system is introduced (e.g., modifications in initial conditions, geometric dimensions, or physical parameters), the computational overhead (in CPU time and RAM) can be excessively or even prohibitively augmented in the case of real-world applications. In this context, ML and deep learning algorithms attempt to replace or accelerate conventional methods such as the method of moments (MoM), the finite-difference time-domain (FDTD) technique, or even some electromagnetic inverse solving approaches [67].

Specifically, concerning the FEM, a variety of efficient ML approaches has been launched, focusing mainly on neural networks and deep learning [68] for the analysis biomechanic applications, which could potentially be extended to treat bioelectromagnetic problems. In light of these considerations, the basic ML procedure involves the following steps:

- *Step 1: Data generation*. The core mesh is generated through the use of widely accepted parameters, accompanied by the necessary statistical bounds, limits, or ranges, and its overall biomechanical behavior is then investigated with the FEM. In this manner and via the appropriate changes and adaptations, instead of the application of the regular computational method to the electromagnetic data, ML schemes can be used to study the behavior of demanding biomedical systems or even suggest means for the improvement of their statistical performance;
- *Step 2: Splitting the data*. The data are split into three sets for training, validation, and testing. This step may be repeated to increase the robustness of the overall system and serve as a trustworthy tool for safe cross-validation;
- *Step 3: Model training*. Using the datasets obtained in step 2, the model is trained according to the initial assumptions or physical conventions;
- *Step 4: Performance evaluation*. The performance metrics primarily utilized in ML approaches are the mean square error and the mean absolute error, which refer to the difference between the calculated and reference quantities of the bioelectromagnetic problem.

Lastly, it should be stressed that although the aforementioned models are, in fact, computationally faster than typical models, they, too, face some challenges. A common problem associated with these algorithms is so-called overfitting, in which the model performs excellently for the given dataset yet poorly when it comes to new data. Furthermore, even without the previous issue, the proposed models may lack the necessary generality and universal applicability, as they can only be trained for a limited number of tasks.

#### 2.2.4. Model Characteristics and Limitations

Bearing in mind the gradually increasing demands associated with the establishment of effective models, this section summarizes the basic applications, computational characteristics, and inevitable limitations of each model for the analysis of bioelectromagnetic data.

The most important merit of analytical models is their generic character, including minimal system overhead and no need for the involvement of any specialized computational software. Moreover, they can be extended or modified to satisfactorily handle a large variety of medical conditions (e.g., thyroid cancer, hypothyroidism, etc.) or serve as reference models when a fast estimation of thyroid condition is required. Nonetheless, due to their simplistic establishment, they cannot support pertinent fine details when it comes to approaching anatomical reality—an issue that makes them inappropriate for sensitive case studies.

Computational models, on the other hand, can be employed for the construction of highly detailed and adjustable-resolution thyroid models. This is, in fact, the most significant reason for their impressive popularity among the researchers in the scientific community. Their most important advantages are summarized as follows:

- *Complex system modeling:* They can manipulate systems that are too complicated for any analytical solution—a fact that is very frequently encountered in biomedical problems.
- *Parametric studies*: The parameters and conditions of the problem can easily be varied in order to investigate a broad range of cases and applications, which is basically impossible in the majority of biomedical problems.
- *Incorporation of theoretical frameworks*: They can be employed to verify theoretical formulations by offering numerical solutions to various demanding cases.

Nevertheless, computational models exhibit some nontrivial shortcomings, namely

- *Computational overhead*: High-fidelity simulations can require considerable computational power, RAM, and CPU time, which may not always be available.
- *Dependence on models*: They are frequently based on certain conventions and approximations, which can reduce their universality and yield errors.
- *Algorithmic limitations*: Their efficiency can be restrained by the methods available, whose inherent artifacts (e.g., numerical dispersion, dissipation, and anisotropy errors or lack of convergence) can deteriorate the levels of accuracy.

Finally, regarding AI and ML implementations, their consistent use can offer new insights into thyroid imaging data, especially when it comes to diagnostics. Being case sensitive, such models can be truly instructive for diagnostic purposes and can provide faster results than computational models. However, their merits are also their weakness. Indeed, they depend on computational or experimental results (used for their training) and are not fit for reference models, since they can only be trained to assess the response under specific variations (e.g., interpolation over a range predefined by training data) and not designed to generically model the thyroid.

Based on the factors discussed herein (summarized in Table 4), it becomes evident that the selection of the appropriate model depends on the problem under study and its parameters. In fact, the ideal approach could involve a combination of both analytical and computational models to attain a comprehensive understanding of the underlying thyroid functions.

## 3. Summary and Future Prospects

Motivated by the concerns raised around the possible implication of human thyroid exposure to radiation and the increasing demand for simulation models of the thyroid, we have provided an instructive survey of the most popular biosensor-based screening techniques, as well as imaging algorithms and models, for the analysis of bioelectromagnetic data. For the sake of comprehension, the bioelectromagnetic models are divided into three large categories based on the evaluated parameters, i.e., volumetric, thermographic, and radiation parameters. In this context, we have, additionally, focused on the more elaborate description of the frequently employed SfM methodology for the efficient design of 3D thyroid models, along with an interesting statistical classification of the related bibliography. Finally, particular attention is drawn to analytical formulations, computational techniques, AI models, and ML techniques for the systematic analysis of bioelectromagnetic thyroid-based data and the precise, full-wave solution of the corresponding electromagnetic problem.

These models and the related methods could enable multi-parametric studies and enrich diagnostic systems for early assessment and treatment of thyroid diseases. The combination of different bioelectromagnetic models and analysis techniques might, eventually, lead to significant improvements in medical equipment, e.g., faster results extraction, higher imaging resolution, and AI-enabled pre-diagnosis. Lastly, the outcomes of the simulations provide helpful insight into the nature of the thyroid, simplifying the design of experimental processes when it comes to thyroid research. This has been exactly the purpose of this survey, namely to offer an elaborate overview of the available methodologies, as well as research suggestions, thereby facilitating the choice of a correct bioelectromagnetic model.

## Figures and Tables

**Table 1:** Operational spectrum and applications of screening techniques.

Screening Technique	Operational Spectrum	Applications
Magnetic Resonance Imaging (MRI)	Radio frequency	Volumetric models and tissue property assessment
Computer Tomography (CT)	X-ray	Volumetric models and tissue property assessment
Positron Emission Tomography (PET)	Gamma rays	Volumetric models and tissue property assessment
X-Ray	X-ray	Volumetric models and tissue property assessment
Thermal Imaging	Infrared	Volumetric models and thermal mapping

**Table 2:** Main parameters for the choice of the solid state sensor.

Device	Measured Parameter	Advantages	Disadvantages
Thermocouple	Voltage	Simplicity, durability, affordability, diversity, self-powered, wide temperature range	Non linearity, low voltage signal, lowest sensitivity, lowest stability reference
Resistance temperature detector	Resistance	Accuracy, stability, linearity	Expensiveness, self heating, low output signal, low absolute resistance, current source required
Thermistor	Resistance	High sensitivity, fast response time, high output signal, 2-wire Ohms measurement	Self heating, non linearity, narrow temperature range, current source required, fragility
Liquid crystal sensor	Color change	Simplicity, durability, resistance sensitivity	Narrow temperature range, low response time, limited number of possible configurations

**Table 3:** Categorization of the related publications based on the preferred method of analysis, the frequency band, and the objective.

Method	Source	Frequency	Objective
Volumetry	[32,34,35,36]	Infrared and X-ray	Reference and diagnostic
Thermography	[32,37,42,43,44,45,46,47]	Infrared, microwave, and X-ray	Reference, diagnostic, and therapeutic
Radiation	[25,27,28,50,51,52,53,54,55]	RF and microwave	Reference, diagnostic, and therapeutic

**Table 4:** Main considerations for the choice of a model.

Model	Applications	Advantages	Disadvantages
Analytical	Diagnostics and reference	Simplicity, speed, computational affordability, and generality	Not case-sensitive
Computational	Diagnostics and reference	Accuracy and generality	Computationally expensive and slow
Artificial Intelligence	Diagnostics	Case sensitivity, accuracy, and speed	Computational expensive and lack of generality

## Data Availability

Data are contained within the article.
